# G‐Quadruplex Formation in a Putative Coding Region of White Spot Syndrome Virus: Structural and Thermodynamic Aspects

**DOI:** 10.1002/cbic.202100064

**Published:** 2021-04-06

**Authors:** Yoanes Maria Vianney, Maria Goretti M. Purwanto, Klaus Weisz

**Affiliations:** ^1^ Institute of Biochemistry Universität Greifswald Felix-Hausdorff Str. 4 17489 Greifswald Germany; ^2^ Faculty of Biotechnology Universitas Surabaya Kalirungkut Str. Surabaya 60293 Indonesia

**Keywords:** G-quadruplexes, NMR spectroscopy, thermodynamics, white spot syndrome virus

## Abstract

White spot disease (WSD) is one of the most devastating viral infections of crustaceans caused by the white spot syndrome virus (WSSV). A conserved sequence *WSSV131* in the DNA genome of WSSV was found to fold into a polymorphic G‐quadruplex structure. Supported by two mutant sequences with single G→T substitutions in the third G_4_ tract of *WSSV131*, circular dichroism and NMR spectroscopic analyses demonstrate folding of the wild‐type sequence into a three‐tetrad parallel topology comprising three propeller loops with a major 1 : 3 : 1 and a minor 1 : 2 : 2 loop length arrangement. A thermodynamic analysis of quadruplex formation by differential scanning calorimetry (DSC) indicates a thermodynamically more stable 1 : 3 : 1 loop isomer. DSC also revealed the formation of additional highly stable multimeric species with populations depending on potassium ion concentration.

## Graphical abstract

**The right spot for white spot**: A G‐quadruplex‐forming sequence identified within the DNA genome of white spot syndrome virus folds into a major and a thermodynamically less stable minor loop isomer. The parallel quadruplex scaffold might constitute a potential target for viral drug development against white spot disease.

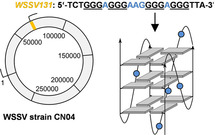

G‐quadruplexes (G4s) are secondary structures of DNA, formed by repeated runs of contiguous guanosine residues. They are widely found throughout different organisms but also occur in viral genomes.[^1^, ^2^] G4s have been shown to be involved in the regulation of gene expression, either acting within the regulatory region or the gene itself.[^3^, ^4^, ^5^] Consequently, formation of G‐quadruplexes in the viral functional genome may contribute to viral mortality and G‐quadruplex‐stabilizing ligands may be employed for viral control.[^6^, ^7^, ^8^, ^9^] The white spot syndrome virus has emerged as one of the most common and most devastating pathogens for farmed crustaceans such as shrimp.[^10^, ^11^] WSSV infects all major shrimp species and has caused huge economic losses in the aquaculture industry worldwide due to the lack of effective treatments. Screening the genome of the white spot syndrome virus (accession number KY827813), we found a conserved sequence with putative G4‐forming ability termed *WSSV131* (Figure 1). Its first G tract is located three bases downstream the template strand of the open reading frame (ORF) WSV131, encoding a putative yet still unknown protein.[^12^, ^13^]

**Figure 1:**
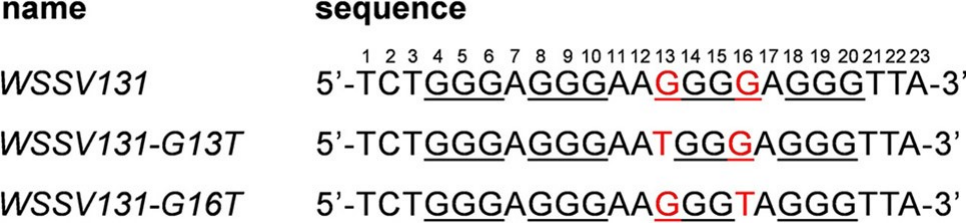
*WSSV131* wild‐type and mutant sequences; G tracts are underlined and positions 13 and 16 marked in red.

The circular dichroism (CD) spectrum of *WSSV131* exhibits positive and negative amplitudes at ∼265 and ∼240 nm, typical for a quadruplex with parallel topology and exclusive homopolar stacking interactions (Figure 2A). The imino proton spectral region of a 1D NMR spectrum for *WSSV131* reveals a set of 12 major imino resonances between 10.5–12.0 ppm characteristic for Hoogsteen hydrogen‐bonded guanines arranged in a G‐tetrad (Figure 2B). However, additional minor species account for ∼30 % of the total population. Polymorphism may arise due to the third tract composed of four G residues to potentially form 1 : 3 : 1 and 1 : 2 : 2 loop isomers. To lock structures to a single loop arrangement, single‐site mutants G13T and G16T mimicking 1 : 3 : 1 and 1 : 2 : 2 loop isomers were evaluated and compared to the wild‐type sequence (Figure 1). CD spectra of the mutants featured highly similar signatures typical of a parallel fold (Figure 2A). Notably, imino proton spectral regions for both mutants indicate a single quadruplex comprising twelve imino resonances with their superposition closely matching native *WSSV131* imino signals with the G13T mutant in excess (Figure 2B). These results suggest the presence of a major 1 : 3 : 1 and a minor 1 : 2 : 2 loop isomer for polymorphic *WSSV131*.

**Figure 2:**
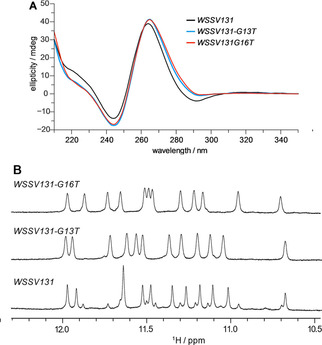
A) CD spectra (20 mM potassium phosphate, pH 7.0, 100 mM KCl, 20 °C) and B) imino proton NMR spectral region (10 mM potassium phosphate, pH 7.0, 25 °C) for *WSSV131* and its G13T and G16T mutants. Note that topologies are conserved under both buffer conditions (Figures S1 and S2 in the Supporting Information).

A more detailed NMR structural analysis of the native *WSSV131* sequence employed standard strategies to assign the major loop isomer. Continuous sugar‐base NOE connectivities are observed between the 5′‐terminal T and the first G‐column, between the fourth G‐column and the 3′ terminus, as well as between guanosines constituting the two central G tracts (Figure 3A). Two prominent crosspeaks in the H8/6‐H1′/H5 spectral region observed at long but also shorter mixing times are identified as A11 H8‐H1′ and C2 H6‐H5 contacts through a ^1^H,^13^C HSQC experiment (Figure S3). Imino‐H8 and imino‐imino NOE contacts identified guanines involved in G‐tetrad formation (Figure 3B, C). There is no indication of any *syn*‐guanosine within the four G tracts in the NOESY spectra in line with the observation of exclusively upfield‐shifted guanine ^13^C8 resonances in the HSQC spectrum characteristic for *anti* conformers (Figure S3). The all‐*anti* G‐quadruplex matches a parallel topology with G‐columns linked by three propeller‐type loops.

**Figure 3:**
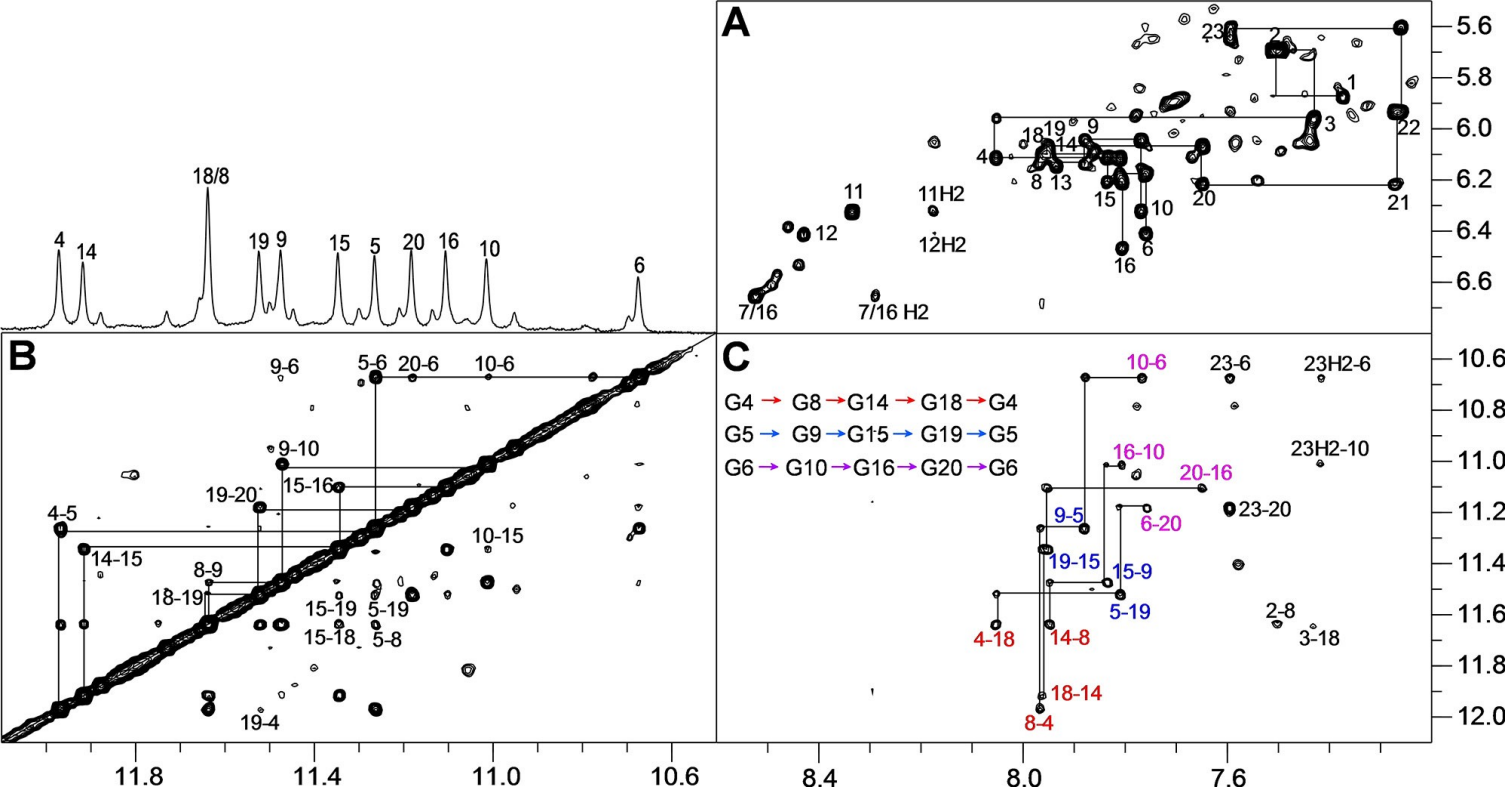
2D NOE spectral regions of *WSSV131* acquired with a 300 ms mixing time at 25 °C. A) H6/8(ω_2_)‐H1′/H5(ω_1_) spectral region with continuous NOE walks along G tracts and 5′‐ and 3′‐overhang sequences. B) Imino–imino crosspeaks with sequential connectivities traced along the G tracts. C) Intra‐ and inter‐tetrad H8(ω_2_)‐imino(ω_1_) crosspeaks; tetrad polarities as determined from intra‐tetrad NOE contacts (marked in red for the 5′‐tetrad, in blue for the central tetrad, and in magenta for the 3′‐tetrad) are given in the inset.

Intra‐tetrad H8‐H1 crosspeaks determine the polarity of the G‐tetrads, that is, the direction when going from H‐bond donor to H‐bond acceptor (Figure 3C). Thus, polarities of 5′‐tetrad, central, and 3′‐tetrad follow G4→G8→G14→G18, G5→G9→G15→G19, and G6→G10→G16→G20, respectively. Polarity is additionally confirmed by typical imino‐imino NOE contacts (Figure 3B). Strong crosspeaks connect sequential Gs within the same G tract. In addition, intra‐tetrad crosspeaks can be seen between neighboring G columns, for example, G10‐G6 and G20‐G6, and inter‐tetrad contacts connect imino protons in adjacent tetrads, such as, G9‐G6 and G10‐G15.

There is also a weak NOE crosspeak between A7 H8 of the first propeller loop and preceding G6 H2′. Likewise, residues of the second propeller loop can be identified through NOE walks based on H8/H6‐H1′/H2′/H3′ contacts from A11 through G13. Another weak sequential contact from G16 H2′ to A17 H8 of the third propeller loop also allows assignment of G16 as being the last residue of the third column (Figure S4). Taken together, experimental findings clearly confirm a major *WSSV131* G4 with parallel topology and a central 3‐nt propeller loop (Figure 4, for a compilation of chemical shifts, see Table S1). A corresponding more detailed 2D NMR spectral analysis on both G13T and G16T mutants confirmed their parallel fold and the same 1 : 3 : 1 loop arrangement for *WSSV131‐G13T* as identified for the major species of the wild‐type sequence (Figure S5 and S6).

**Figure 4:**
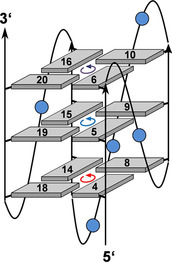
Topology of the *WSSV131* major quadruplex species with tetrad polarities indicated and loop residues represented by circles; residues of the G‐core are numbered.

Initial UV melting studies on the *WSSV131* G13T and G16T mutants, each forming a single loop isomer, revealed slow kinetics of (un)folding at 10 mM K^+^ whereas at 120 mM K^+^ melting profiles shifted to temperatures too high for the observation of a well‐defined high‐temperature baseline within the temperature window accessible (not shown). To nevertheless obtain information on their folding thermodynamics, quadruplex formation was analyzed by DSC in a pressurized cell allowing sample heating up to 110 °C without irreversible DNA decomposition. Thermodynamic equilibrium upon heating was verified for both sequences by a match in melting profiles determined with two different heating rates (not shown). After proper baseline correction, DSC data were fitted with a non‐two‐state model assuming negligible changes in molar heat capacity Δ*C*_p_ ^[14]^ to yield *T*_m_ as well as calorimetric and van′t Hoff molar enthalpies ΔH∘cal and ΔH∘vH (Figure 5, Table S2).^[15]^

**Figure 5:**
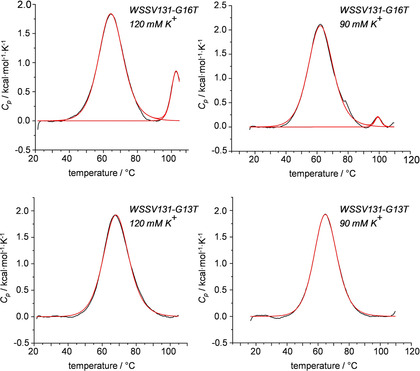
Representative DSC thermograms with a 0.5 °C/min heating rate of *WSSV131* sequences (50 μM). Melting profiles for *WSSV131‐G16T* (top) in the presence of 120 mM K^+^ (left) and 90 mM K^+^ (right). Melting profiles for *WSSV131‐G13T* (bottom) in the presence of120 mM K^+^ (left) and 90 mM K^+^ (right). Fitted curves based on a non‐two‐state model with ΔH∘cal ≠ΔH∘vH are shown in red.

In a buffer solution with 120 mM K^+^ the major *WSSV131‐G13T* quadruplex exhibits a *T*_m_ of 68.1 °C, 3.4 °C higher than that of the *WSSV131‐G16T* mutant. The higher melting 1 : 3 : 1 propeller loop arrangement of *WSSV131‐G13T* agrees with systematic studies on the length of propeller loops, reporting a propensity of short first and third loops with a longer central loop.^[16]^ Interestingly, the less favored *WSSV131‐G16T* quadruplex shows another high‐temperature transition centered at 103 °C but not fully completed within the experimental temperature range. To shift transitions towards lower temperatures, DSC measurements were also performed in solutions with 90 mM K^+^. Again, in contrast to *WSSV131‐G13T* the G16T mutant exhibits an additional high‐temperature transition shifted to 99.3 °C but with noticeably reduced height. Based primarily on gel electrophoresis and size exclusion chromatography, high‐melting multimeric species have previously been suggested to coexist in particular for parallel‐stranded quadruplexes with short loops.[^17^, ^18^, ^19^, ^20^] However, they have not been observed and characterized by calorimetric methods so far. Taking into account a growing population and faster folding of multimers with increasing K^+^ concentration, ΔH∘cal for the monomer transition at lower temperature is expected to be significantly underestimated in line with a ΔH∘vH /ΔH∘cal >1 in a 120 mM K^+^ buffer. On the other hand, the small population of multimers at 90 mM K^+^ does hardly compromise ΔH∘cal for *WSSV131‐G16T*, yielding a ΔH∘vH /ΔH∘cal ratio of about 1 in agreement with a two‐state melting transition for the monomer. Because ΔH∘vH is independent of concentration and only depends on the shape of the DSC curve, ΔH∘vH is expected to provide a reliable value for the enthalpy of (un)folding given a two‐state transition under equilibrium conditions. With a ΔH∘vH of −47.0 and −46.1 kcal/mol for *WSSV131‐G13T* and *WSSV131‐G16T* at 120 mM K^+^ as well as −45.9 and −42.9 kcal/mol at 90 mM K^+^, folding of the more stable G13T mutant seems more exothermic by 1 and 3 kcal/mol. It should also be noted that despite observation of only one DSC transition, ΔH∘vH /ΔH∘cal was found to be >1 under both salt conditions for *WSSV131‐G13T*. This strongly suggests formation of corresponding multimers with even higher thermal stability when compared to those of *WSSV131‐G16T*, escaping their detection in the DSC experiment. Indeed, formation of higher‐order assemblies for both G16T and G13T mutant sequences are also indicated by native gel electrophoresis experiments in a 90 mM K^+^ buffer (Figure S7).

Taken together, a well‐defined parallel quadruplex, highly stable under physiological salt conditions, is formed in a sequence located downstream of a putative coding region in the WSSV viral genome. Such a G4 could possibly be used to regulate viral gene expression and offers the opportunity to ultimately control WSSV infection through the use of G4‐binding and G4‐stabilizing ligands. Detection and characterization of such G4‐prone sequences in the viral genome open new avenues for the target design of antiviral drugs directed against WSSV in crustacean farming.

## Conflict of interest

The authors declare no conflict of interest.

## Supporting information

As a service to our authors and readers, this journal provides supporting information supplied by the authors. Such materials are peer reviewed and may be re‐organized for online delivery, but are not copy‐edited or typeset. Technical support issues arising from supporting information (other than missing files) should be addressed to the authors.

Supplementary
