# Supplementary material for: G‐Quadruplex Formation in a Putative Coding Region of White Spot Syndrome Virus: Structural and Thermodynamic Aspects
Source: Chembiochem. 2021 Apr 6;22(11):1932–5. doi: 10.1002/cbic.202100064 (PMC8252560; doi:10.1002/cbic.202100064)
Supplement: Supplementary file 1 — Supplementary [file CBIC-22-1932-s001.pdf]

ChemBioChem

Supporting Information

G-Quadruplex Formation in a Putative Coding Region of White Spot Syndrome Virus: Structural and Thermodynamic Aspects

Yoanes Maria Vianney, Maria Goretti M. Purwanto, and Klaus Weisz*

Experimental Section

Sample preparation. Oligonucleotides were purchased from TIB MolBiol (Berlin, Germany), further purified by ethanol precipitation, dried, and dissolved in water. The concentration was measured through their absorbance at 260 nm at 80 ºC using the extinction coefficient provided by the manufacturer. After drying, oligonucleotides were resuspended for NMR assignments in a 10 mM potassium phosphate buffer, pH 7.0, with 90% H_2O/10% D_2O. For other experiments, a 20 mM potassium phosphate buffer with 100 mM KCl, pH 7.0, was used unless otherwise stated in the text. Prior to usage, samples were annealed by heating to 90 ºC for 5 minutes followed by slow cooling to room temperature.

CD spectroscopy. CD spectra were recorded on samples (5 µM) in 1-cm quartz cuvettes with a Jasco J-810 spectropolarimeter equipped with a Peltier thermostat (Jasco, Tokyo, Japan). Spectra were acquired with a wavelength range of 210-350 nm, a 1 nm bandwidth, a 50 nm/min scanning speed, 4 s response time and 5 accumulations. All spectra were recorded at 20 ºC and finally blank-corrected.

NMR spectroscopy. NMR spectra were recorded with a Bruker Avance 600 MHz spectrometer equipped with an inverse $^{1}H/^{13}C/^{15}N/^{19}F$ quadruple resonance cryoprobehead and z-field gradients. Proton chemical shifts were referenced to the water chemical shift at 25 ºC while carbon chemical shifts were referenced to DSS by an indirect referencing method. 2D ^{1}H-^{1}H NOESY experiments were performed with a 300 ms and 80 ms mixing time. 2D ^{1}H-^{13}C HSQC experiments were recorded with 7500 Hz in the indirect dimension for recording aromatic carbon resonances. All spectra were acquired at 25 ºC with oligonucleotide concentrations of about 0.3 mM.

Differential Scanning Calorimetry (DSC). DSC experiments were carried out with a VP-DSC instrument (Malvern Instruments, United Kingdom). The oligonucleotide solution (50 µM) was heated at a heating rate of 0.5 $ºC{\cdot}min^{-1}$ (three independent measurements) and 0.25 $ºC{\cdot}min^{-1}$ (two independent measurements) with a pre-equilibration time of 15 min. Buffer vs. buffer scan data were taken by refilling the sample cell in the same running cycle and subtracted from the sample data. After cubic baseline correction, data were fitted with a non-two-state model assuming ΔC_p = 0 $kcal{\cdot}mol^{-1}{\cdot}K^{-1}$ to obtain T_m, $\Delta H°_{cal}$, and $\Delta H°_{vH}$.

Native Gel Electrophoresis. 100 µM of annealed oligonucleotide in a 20 mM potassium phosphate buffer, pH 7.0, supplemented with 70 mM KCl were mixed with glycerol-buffer (4:6) in a 1:1 v/v ratio. Electrophoresis experiments were performed on a 15% polyacrylamide gel (acrylamide:bis-acrylamide 19:1). Samples (1 nmol per lane) were loaded and separation was performed at room temperature in TBE buffer, pH 8.3, supplemented with 90 mM KCl. Gels were stained with 5 µM thiazole orange.

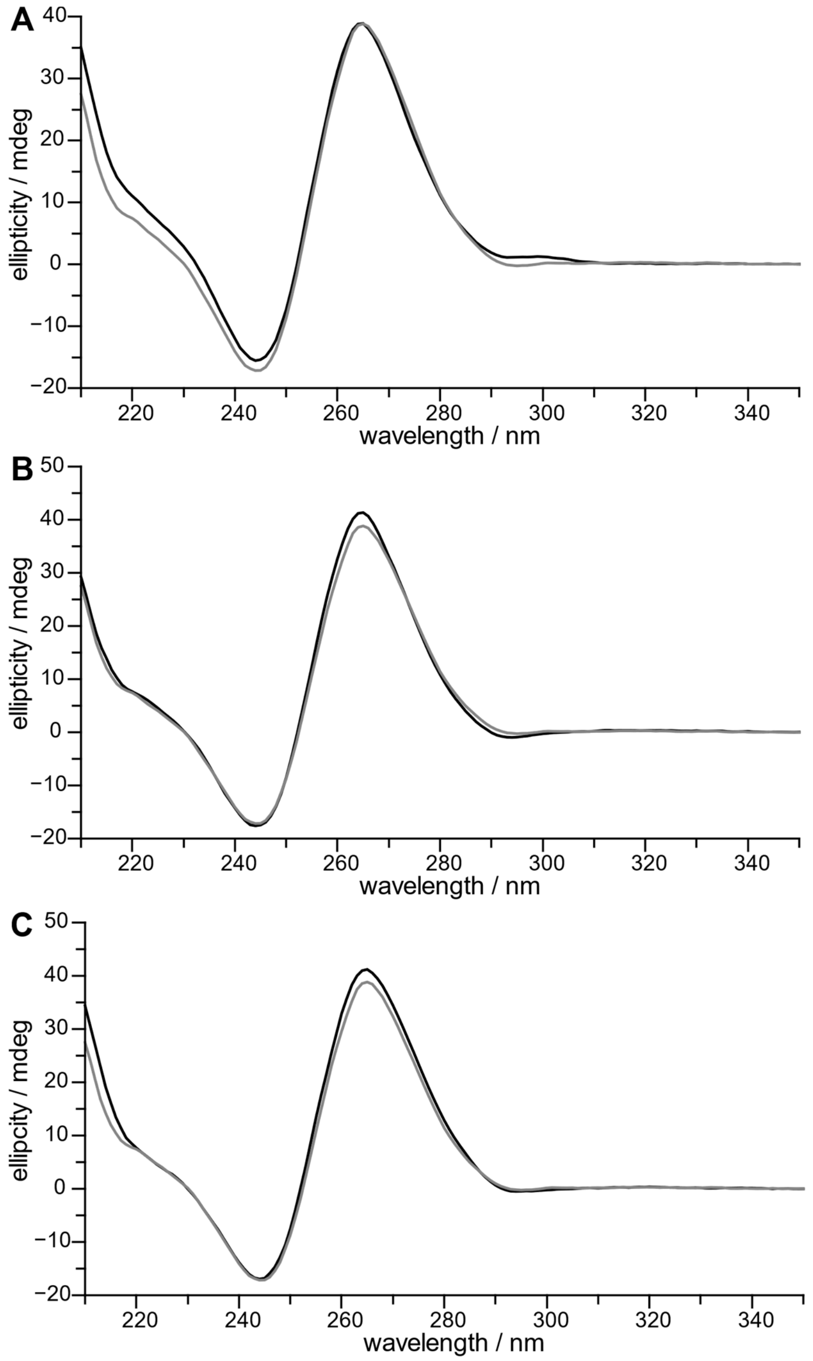

Figure S1. CD spectra for (A) *WSSV131*, (B) *WSSV131-G13T*, and (C) *WSSV131-G16T* in 20 mM potassium phosphate buffer + 100 mM KCl, pH 7.0 (black), and 10 mM potassium phosphate buffer, pH 7.0 (grey). No significant changes are observed for the CD profiles.

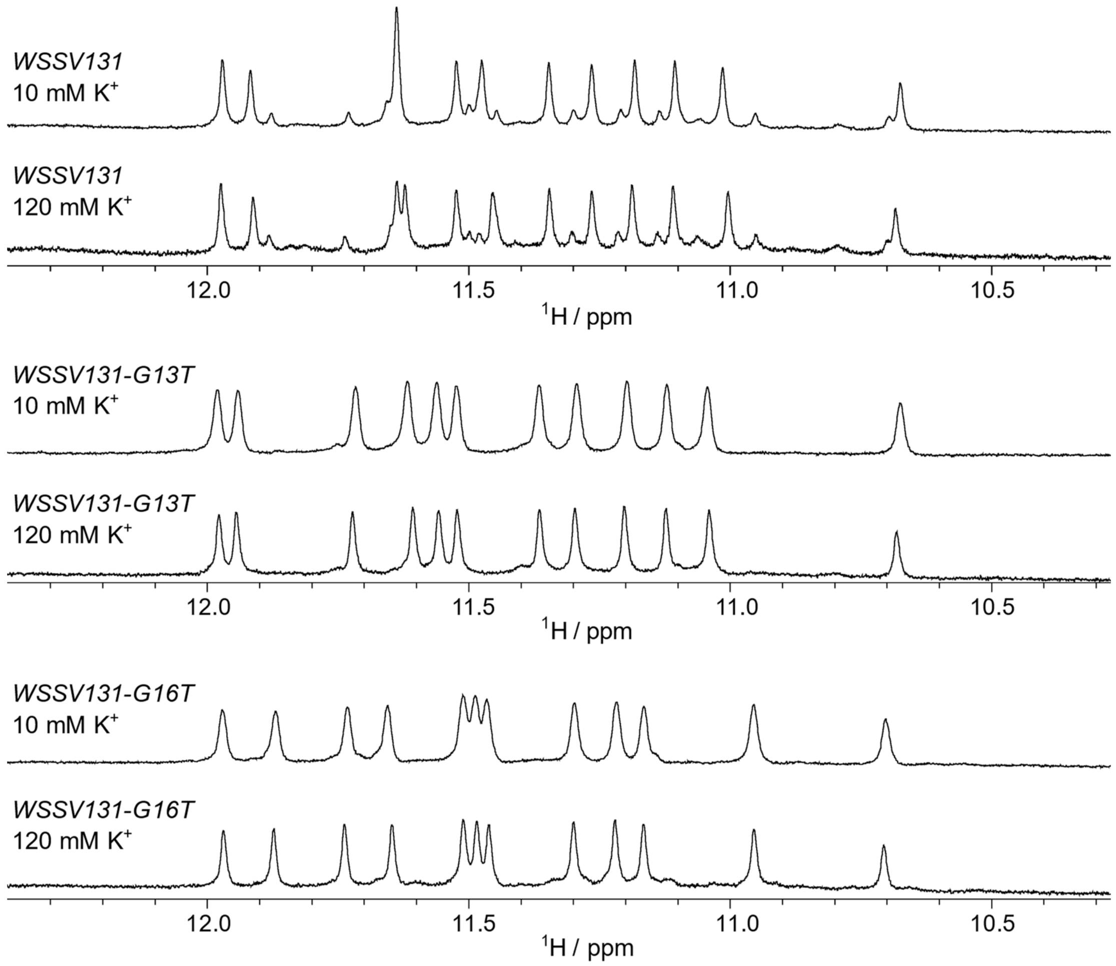

Figure S2. Imino proton spectral region of *WSSV131* (top), *WSSV131-G13T* (center), and *WSSV131-G16T* (bottom) variants (0.3 mM) in 10 mM potassium phosphate buffer, pH 7, and in 20 mM potassium phosphate buffer + 100 mM KCl, pH 7.0.

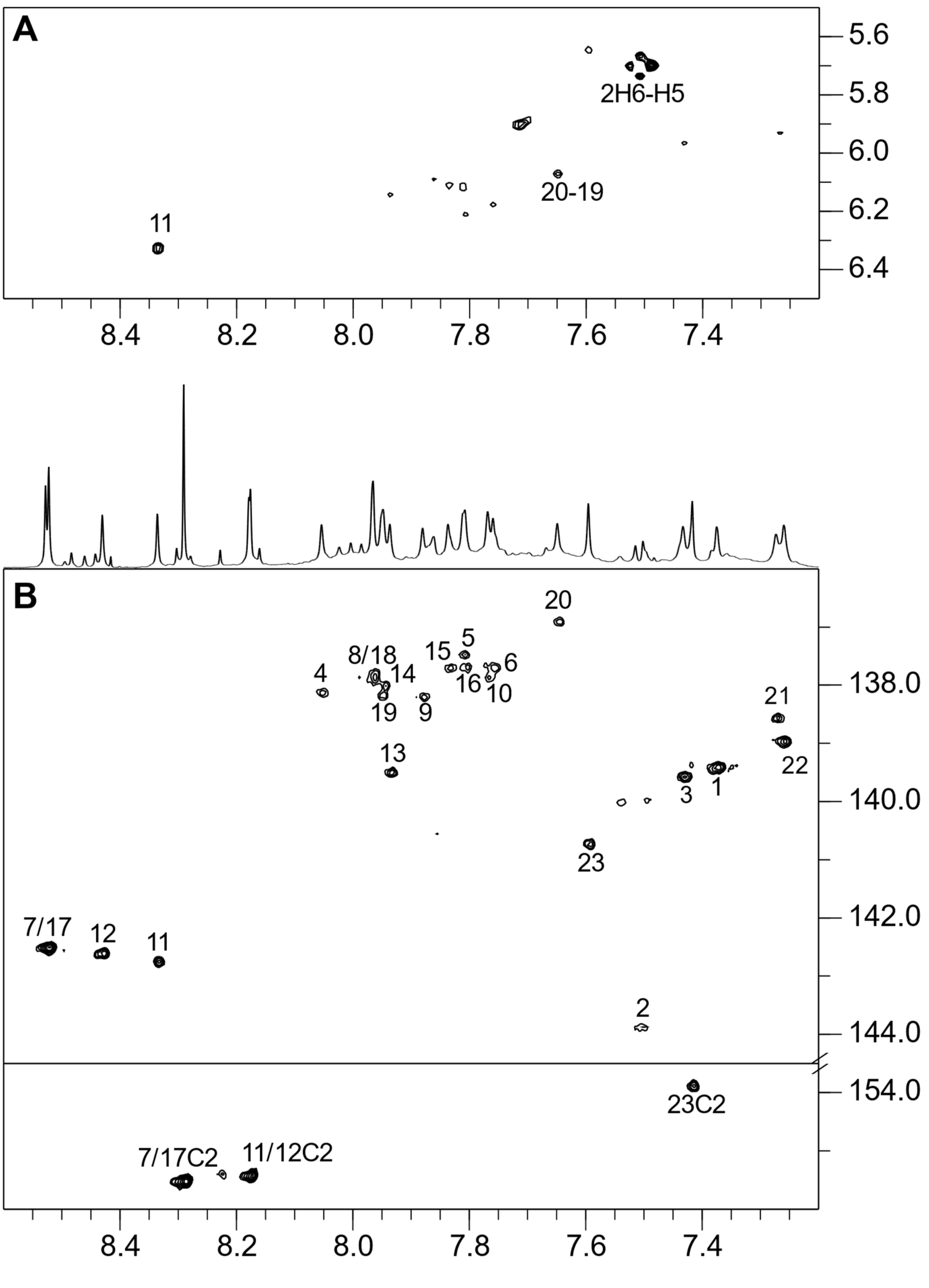

Figure S3. (A) H6/H8 (ω_2) - H1'(ω_1) NOESY spectral region of *WSSV131* at 80 ms mixing time. (B) H6/H8/H2(ω_2) - C6/C8(ω_1) spectral region of a ^{1}H-^{13}C HSQC spectrum of *WSSV131* (0.3 mM). Both spectra were recorded at 25 ºC in 10 mM potassium phosphate buffer, pH 7.

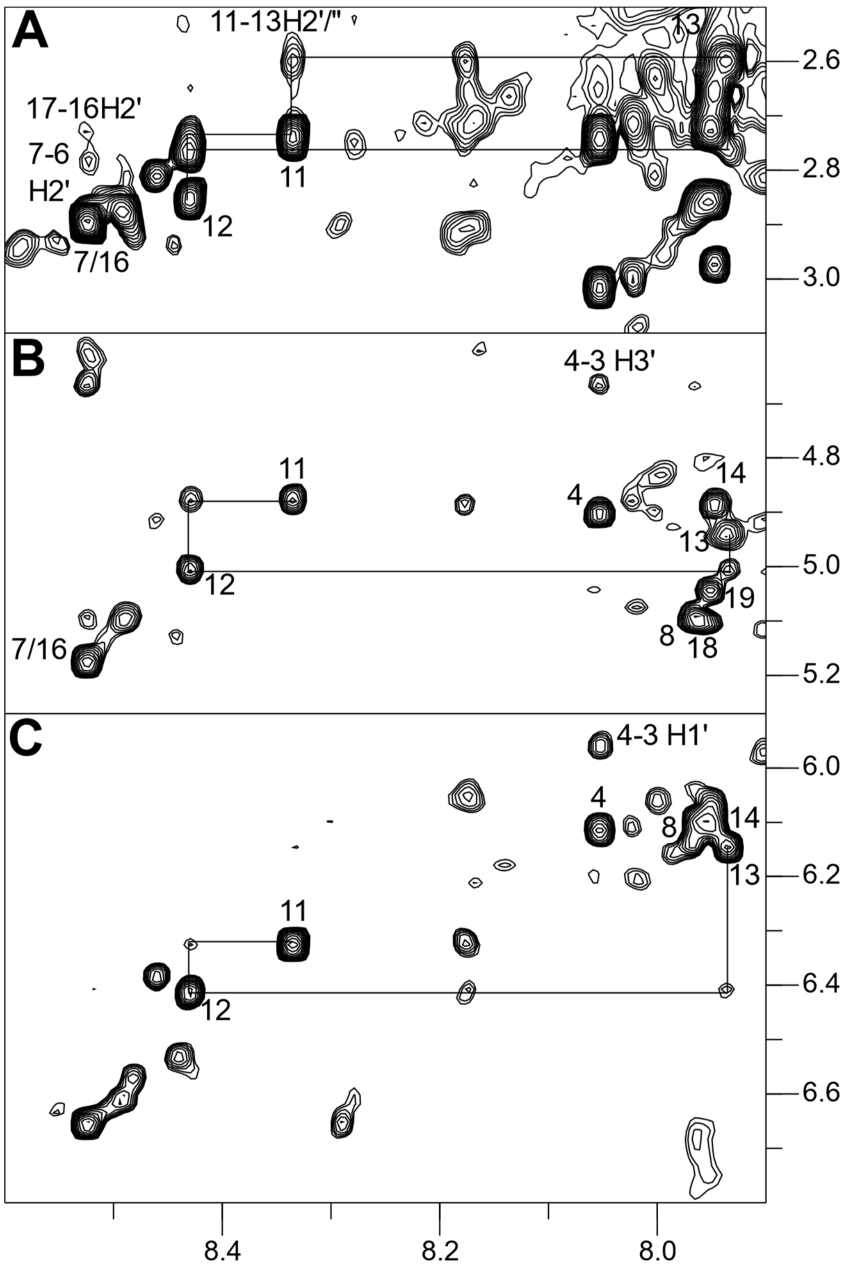

Figure S4. NOESY spectral regions of *WSSV131* showing H8/H6 - sugar contacts involving adenosine in the propeller loop. (A) H8/H6(ω_2) - H2'/H2"(ω_1) region, (B) H8/H6(ω_2) - H3'(ω_1) region, and (C) H8/H6(ω_2) - H1'(ω_1) region. Spectra were recorded at 25 ºC in 10 mM potassium phosphate buffer, pH 7.

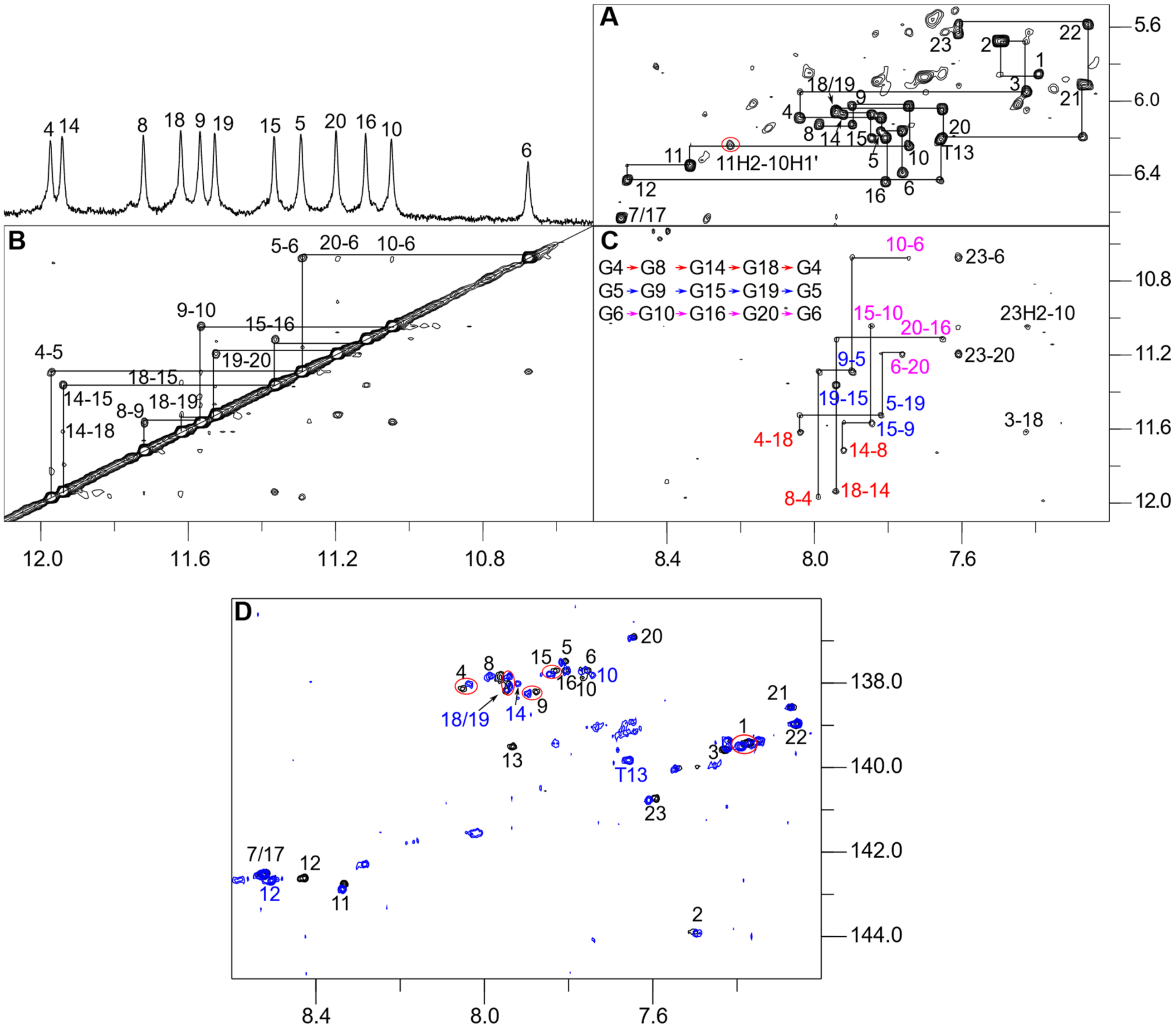

Figure S5. (A-C) 2D NOE spectral regions of *WSSV131-G13T* (0.3 mM) acquired with a 300 ms mixing time. (A) H6/8(ω_2) - H1'/H5(ω_1) spectral region with NOE walks along G-tracts and 5'- and 3'-overhang sequences. Loop isomer 1:3:1 is identified through NOE contacts from A11 H2 to G10 H1' (circled in red). (B) Imino - imino cross-peaks with sequential connectivities traced along the G-tracts. (C) Intra- and inter-tetrad H8(ω_2) - imino(ω_1) cross-peaks; tetrad polarities as determined from intra-tetrad NOE contacts (marked in red for the 5'-tetrad, in blue for the central tetrad, and in magenta for the 3'-tetrad) are given by the inset. (D) H6/H8(ω_2) - C6/C8(ω_1) spectral region of a ^{1}H-^{13}C HSQC spectrum of *WSSV131* (black) and *WSSV131-G13T* (0.3 mM, blue). Notably, most cross-peaks superimpose except for exchanged residue 13, indicating the same fold with a 1:3:1 loop arrangement. Both spectra were recorded at 25 ºC in 10 mM potassium phosphate buffer, pH 7.

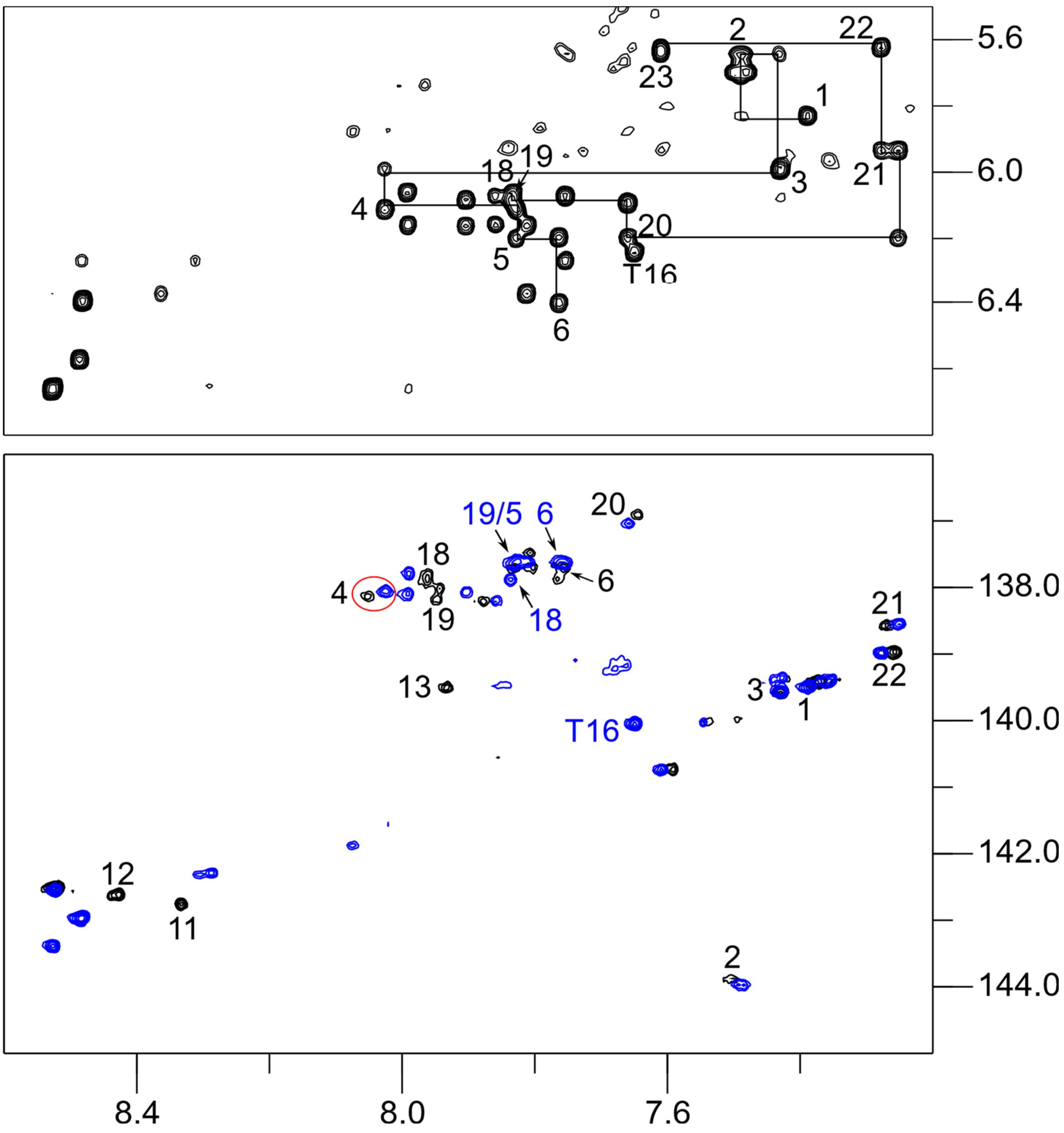

Figure S6. (Top) H6/H8(ω2) - H1'(ω1) 2D NOE spectral region of *WSSV131-G16T* (0.3 mM) acquired with a 300 ms mixing time. (Bottom) H6/H8(ω2) - C6/C8(ω1) spectral region of a ^{1}H-^{13}C HSQC spectrum of *WSSV131* (black) and *WSSV131-G16T* (0.3 mM, blue). More pronounced chemical shift differences are noticeable for several residues. Both spectra were recorded at 25 ºC in 10 mM potassium phosphate buffer, pH 7.

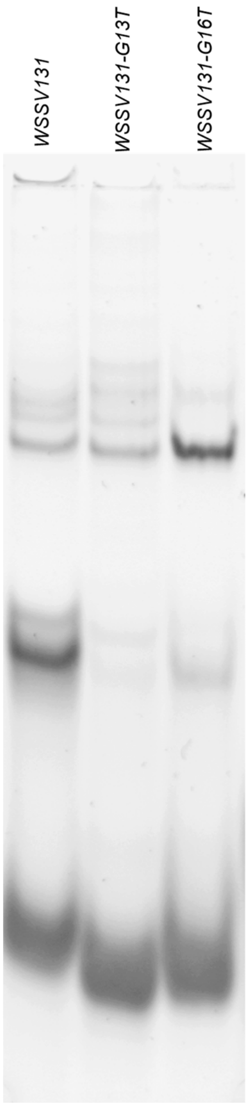

Figure S7. Non-denaturing polyacrylamide gel electrophoresis of *WSSV131* variants in TBE buffer supplemented with 90 mM KCl. Fast, moderate, and slow migrating bands are suggested to be due to monomer, dimers, and multimers.

Table S1. Chemical shifts (ppm) for the major quadruplex of *WSSV131*[a]

	Imino	H6/H8	H5/H2/Me	H1'	H2'	H2"	H3'	C6/C8
T1	n.d.	7.37	1.64	5.88	1.96	2.18	4.46	139.42
C2	-	7.50	5.70	5.69	1.89	2.31	4.61	143.89
T3	n.d.	7.43	1.55	5.96	2.13	2.40	4.70	139.58
G4	11.97	8.05	-	6.11	2.74	3.02	4.93	138.14
G5	11.26	7.81	-	6.18	2.65	2.93	5.04	137.48
G6	10.68	7.76	-	6.41	2.78	2.60	5.12	137.72
A7	-	8.52	8.29	6.66	2.90	2.90	5.21	142.52
G8	11.64	7.97	-	6.14	2.46	2.89	5.13	137.89
G9	11.47	7.88	-	6.05	2.61	2.76	4.98	138.21
G10	11.01	7.77	-	6.32	2.61	2.57	4.92	137.88
A11	-	8.33	8.18	6.33	2.74	2.74	4.91	142.78
A12	-	8.43	8.17	6.41	2.85	2.77	5.04	142.64
G13	n.d.	7.94	-	6.12	-[b]	-[b]	4.98	139.51
G14	11.92	7.95	-	6.12	2.66	2.98	4.92	138.02
G15	11.35	7.84	-	6.21	2.73	2.95	5.04	137.71
G16	11.10	7.81	-	6.47	2.73	2.61	5.12	137.70
A17	-	8.52	8.29	6.66	2.90	2.90	5.21	142.52
G18	11.64	7.96	-	6.10	2.45	2.87	5.13	137.86
G19	11.52	7.95	-	6.07	-[b]	2.73	5.07	138.18
G20	11.18	7.65	-	6.22	2.59	2.80	4.95	136.91
T21	n.d.	7.27	1.55	5.94	2.09	2.37	4.74	138.57
T22	n.d.	7.26	1.62	5.61	1.66	2.14	4.44	138.97
A23	-	7.59	7.42	5.65	2.05	2.26	4.28	140.74

[a]At 25 °C in 10 mM potassium phosphate buffer, pH 7. [b]Ambiguous.

Table S2. DSC-derived thermodynamic parameters for the folding of *WSSV131* sequences.[a]

sequence	T_m (°C)	ΔH^o_{cal} (kcal/mol)[b]	ΔH^o_{vH} (kcal/mol)	T_m (°C)	ΔH^o_{cal} (kcal/mol)[b]	ΔH^o_{vH} (kcal/mol)
	20 mM KP_i (pH 7.0) + 70 mM K^+			20 mM KP_i (pH 7.0) + 100 mM K^+		
WSSV131-G13T	65.0 ± 0.1	-37.6 ± 0.9	-45.9 ± 0.3	68.1 ± 0.2	-37.2 ± 0.8	-47.0 ± 0.4
WSSV131-G16T[c]	61.9 ± 0.5	-42.6 ± 2.1	-42.9 ± 0.9	64.7 ± 0.3	-35.9 ± 0.7	-46.1 ± 0.7
	99.3 ± 0.2	-0.8 ± 0.2	-230.6 ± 30	102.7 ± 0.3	---[e]	---[e]

[a]Average value with standard deviation from three independent measurements. [b]Apparent calorimetric molar enthalpy for a corresponding transition if $c_{DNA} = c_{DNA,total}$. [c]Two transitions observed. [d]Three transitions observed. [e]Not definable due to incomplete melting.
